# Interplay of self-care, self-efficacy, and health deviation self-care requisites: a study on type 2 diabetes patients through the lens of Orem’s self-care theory

**DOI:** 10.1186/s12875-024-02276-w

**Published:** 2024-01-31

**Authors:** Ghorbanali Jennat Fereidooni, Fazlollah Ghofranipour, Fatemeh Zarei

**Affiliations:** Department of health education and health promotion, Faculty of Medical Sciences, Tarbiat Modares University, Theran, Iran

**Keywords:** Self-care, Self-efficacy, Health Deviation Self-Care requisites, Type 2 diabetes Orem’s self-care theory

## Abstract

**Background:**

This study aimed to examine the relationship between Self-Care, Self-Efficacy, and Health Deviation Self-Care Requisites in patients with type 2 diabetes based on Orem’s Self-Care Theory.

**Methods:**

The research involved 341 patients with type 2 diabetes in Rasht, Iran, using a descriptive-analytical cross-sectional design. The data collection included questionnaires assessing Self-Care Behaviors, Self-Efficacy, and Health Deviation Self-Care Requisites based on Orem’s model.

**Results:**

Demographic factors such as gender, marital status, employment, education, age, duration of disease, and oral treatment and insulin had no consistent effect on self-care behaviors. Self-efficacy was a key factor influencing Self-Care Behaviors in diabetic patients. There was a strong and direct correlation between Self-Care Behaviors and Self-Efficacy, indicating the role of individuals’ confidence in managing diabetes. Health Deviation Self-Care Requisites had both positive and negative correlations with different domains of Self-Care Behaviors.The physical exercise construct of self-efficacy was the most significant predictor of Self-Care Behaviors.

**Conclusions:**

This study provides valuable insights into the complex relationship between Self-Care, Self-Efficacy, and Health Deviation Self-Care Requisites in patients with type 2 diabetes. The findings underscore the importance of addressing Self-Efficacy and specific self-care domains, such as physical activity and foot care, in diabetes management strategies. This research contributes to the existing knowledge base and may inform healthcare professionals and policymakers in developing targeted interventions to improve self-care practices in diabetic patients.

## Introduction

Diabetes is a chronic condition that can be managed but not cured. The effectiveness of diabetes treatment largely depends on the patient’s self-care, as the patient is responsible for more than 95% of diabetes care [1]. Self-care is a proactive and practical process that a person performs to maintain and enhance one’s health, prevent and treat illness, and avoid the short- and long-term complications of disease [2]. The literature suggests that patients who engage more actively in self-care achieve better health outcomes [2, 3]. Self-care in diabetic patients involves various aspects, such as monitoring and regulating blood glucose level, adhering to medication, exercising and being physically active, following nutrition and dietary guidelines, caring for the feet, quitting smoking, and adopting other healthy behaviors [4]. Experts recommend that modifying lifestyle-related behaviors, such as dietary changes and physical activities, are among the first-line interventions for diabetes. Therefore, disease management and self-care behaviors (SCB) are essential for controlling diabetes, especially type 2 diabetes, in the current medical era [5]. Several studies in Iran show that, like other patients with chronic diseases, diabetics also have poor self-care, which results in high costs for the patients and the health care system [6–8]. In individuals with diabetes, it is imperative to prioritize self-care strategies to mitigate potential complications and ensure an optimal quality of life [9].

Orem’s self-care model is one of the most comprehensive self-care theories that presents an appropriate clinical guideline for planning and implementing self-care principles [10]. Orem believes that self-care consists of activities a person performs to maintain or promote one’s life, health, well-being, and also to prevent and cure one’s disease. The main philosophy behind Orem’s model is to prepare the patient for taking responsibility for one’s own care. She believes that individuals are capable of taking care of themselves [11]. Based on this model, self-care is an acquirable behavior that can meet many of the patients’ needs during illness or health deviation [12].

The requisite to presenting an effective solution is to find factors that affect SCB. Among studies conducted on factors related to SCB in diabetes, self-efficacy holds a special place and is an important predictor of self-care behavior [13–15]. Self-efficacy is the belief that one has in oneself, wherein a specific behavior is adopted and its expected results are also achieved. Self-efficacy affects one’s motivation and encourages one to try and persevere in a behavior [16]. Several studies have indicated that when an individual has a high perception of one’s ability in handling and controlling diabetes, then it is more likely that s/he participates in self-care activities and subsequently has a better quality of life [17–19]. Self-efficacy is a key element of self-empowerment, and can be developed through a set of meaningful, relevant and successful experiences [20]. To effectively manage diabetes, a patient should undergo essential training and acquire the relevant knowledge and skills. Thus, empowering the patient and supporting self-care through education are the keys to controlling diabetes [21].

The literature on self-care behaviors (SCB) and self-efficacy in patients with type 2 diabetes shows inconsistent findings. Some studies have found a high level of SCB and a strong correlation between self-efficacy and self-care [22–24], while others have reported a low or moderate level of SCB and a weak or non-significant correlation between self-efficacy and self-care [25–27]. Moreover, the rates of SCB, self-efficacy and health deviation self-care requisites (HDSCR) vary across different studies [28–30]. Considering the importance of diabetes management and the gaps in the existing literature, the current study aimed to examine the relationship between SCB, self-efficacy, and HDSCR in patients with type 2 diabetes, using Orem’s self-care model as a theoretical framework.

## Method

### Study design, setting and location

A descriptive – analytical cross-sectional study was conducted. The main objective of this study was to determine the current status and association between SCB, self-efficacy and HDSCR of patients with type 2 diabetes based on Orem’s model.

### Structure of primary health care (PHC) system in Iran

The PHC system in Iran consists of a network of health facilities that serve the rural and urban populations. In each village or group of villages, there is a health-house, where a trained health care provider called Behvarz (Multi - purpose health care worker) takes care of 1200 people. The health-houses are the first point of contact between the health system and the families. The bigger villages also have rural health centers, where a physician and a team of up to 10 health workers handle more complicated health issues. Each rural health center serves about 7000 people. In the cities, health posts and health centers offer similar services as the health-houses and rural health centers. The district health centers manage this network, under the guidance of medical sciences universities. Every province has at least one Medical Sciences University. This study was conducted on patients with type 2 diabetes attending health-houses in Rasht city, Iran.

### Participant recruitment and inclusion/exclusion criteria

The inclusion criteria were, a medical history of physician-confirmed diabetes for more than 1 year, residing in the region for another year, the ability to read and write, drug consumption, age > 30 years, absence of the complications of diabetes, absence of other diseases. The exclusion criteria were: reluctance to continue collaboration for any reason, the onset of other illnesses, and any physical or mental disorders.

### Sampling and sample size

Random multi-stage cluster sampling was done; out of 32 comprehensive health service centers in Rasht, 9 centers were randomly selected, and from each center, 3 health houses (a total of 27 health houses) were randomly chosen. Sampling in healthcare houses was conducted randomly and in proportion to the determined sample size from the 1809 active patient records in each health house. Eligibility examination was conducted on 1809 patients from 27 health houses. Of those, 1125 patients were included, while 684 were excluded. A total of 341 patients completed the questionnaire and were ere analyzed. After listing on the basis of inclusion criteria, 341 patients were randomly selected and included in the study after informed consent was taken. Based on Khosrovan et al’s study [31] titled ‘Examining self-care capacity in diabetic women with peripheral neuropathy and related needs based on Orem’s self-care model’, the mean and standard deviation of the self-care capacity score was 40.29 ± 13.27. Given 5% Types 1 error and a precision of 1.5 from the mean of self-care capacity using the following formula, the required sample size was calculated at 301 individuals. Moreover, considering a 10% design effect in the cluster sampling, and the conditions of the sampling site and the volunteering of the selectees’ companions, eventually, 341 individuals participated in the study.

$$\text{N}=\frac{{\left({\varvec{Z}}_{1-\frac{\varvec{\alpha }}{2}}\right)}^{2}{\varvec{\sigma }}^{2}}{{\varvec{d}}^{2}} = \frac{{1.96}^{2}{\times 13.27}^{2}}{{1.5}^{2}}=301$$

### Data collection instrument

Data was collected using three questionnaires. These will be discussed in detail below.

### Summary of diabetes self-care activities measure (SDSCA)

This questionnaire examines the self-care activities of diabetes patients. It includes 13 questions about self-activities such as, ‘diet’, ‘physical activity’, ‘blood sugar control’, ‘foot care’ and ‘drug consumption’. A score of 0–7 is considered for each question. The minimum and maximum scores are 0 and 7 and the score range is from 0 to 91. The content validity of the scale was approved by an expert panel (10 specialists). The content validity index (CVI) and content validity ratio (CVR) were 0.92 and 0.90, respectively [32].

### Orem’s self-care needs assessment questionnaire

This questionnaire has been used to assess patients’ educational needs in different fields of information, such as, disease and its complications, diet, activity, and Oral treatment and insulin The questionnaire consists of 50 questions which are answered by interviewing the patient. There are two options for each question, “I know” which scores zero, and “I don’t know” which scores 1. The total score attainable is 50. The content validity of the scale was approved by an expert panel (10 specialists). The CVI and CVR were 0.98 and 0.90, respectively [32].

### Self-efficacy questionnaire/scale

In this study, we used this scale to examine the patients’ self-efficacy in self-care. The questionnaire comprises 8 items that are scored based on a 4-item Likert scale; ranging from “I’m completely certain” (4 points) to “I’m not certain at all” (1 point). The mean scores obtained are classified into proper (3.1-4), average (2.1-3), and poor (1–2) categories. The content validity of the tool was approved by an expert panel (10 specialists). Both the CVI and CVR were 1 [15].

### Procedure

The “Sib System” was designed and implemented for the purpose of registering, maintaining, and updating the electronic health records of Iranians. It is connected to the Integrated Health System (HIS) of Iran in each province. The data of participants from health centers in Gilan province, Rasht city, were also obtained from the Sib System of Rasht. After extracting the list of patients from Rasht’s health houses’ electronic records (SIB), and also taking into account the inclusion criteria and random selection of patients (proportionate to each health house’s sample size), the list of selected patients was handed over to the health workers. After arranging with and inviting the patients over to the health houses on a specific day, the researcher visited the health house and completed the questionnaires one by one through interviews. For those patients who were unable to attend the health house, the health worker would arrange a time when the researcher could visit the patient’s house and complete the questionnaire. Patients’ reluctance to cooperate due to the distance from the health houses to their residence, and the fact that most patients were farmers and did not have enough time to attend the health houses to complete the questionnaire. The data collection process was conducted meticulously by the first researcher (GJF), who personally administered and collected the questionnaires. This hands-on approach ensured the completeness of the data, resulting in no missing information in our dataset. The process of questionnaire completion and data collection commenced on March 22, 2021, and spanned a duration of over two months, concluding on May 31, 2021.

### Outcome, predictor, and confounder variable

The outcome variable was “Self-Care Behavior”. The predictor variables included Health Deviation Self-Care Requisites, Self-Efficacy in Diet, Self-Efficacy in Physical Activity, Self-Efficacy in Blood Glycemic Control, Self-Efficacy in Foot Care, and Self-Efficacy in Medication Compliance. Potential confounders, such as age, gender, duration of disease, Oral treatment and insulin, Income, BMI, and History of training, were controlled for in the analysis to ensure the observed associations were not due to these factors. It should be clarified that “Training History” in our study refers to whether the participant type 2 diabetic patients have received any form of diabetes education or training in the past. This could include information about managing their condition, understanding their medication, diet, and lifestyle changes, among other things.

### Addressing potential bias

Several efforts to address potential sources of bias in our study were made:

- **Randomization:** We employed a random sampling strategy to select our study centers and health homes. Specifically, we randomly selected 9 centers from a total of 32 in Rasht city. From these selected centers, we further randomly chose 3 health homes (27 in total). The sample size was proportional to the active files of patients in each health home, ensuring a fair representation.
- **Tool Validation:** We validated our survey questionnaire to ensure its reliability. It received high scores for Content Validity Index (CVI) and Content Validity Ratio (CVR), indicating its suitability for our study.
- **Personal Administration of Questionnaires:** To minimize errors that could arise from self-administration, the researcher personally completed the questionnaires with the participants.

### Ethical considerations

A license was obtained from Tarbiat Modares University’s ethical committee (IR.MODARES.REC.1401.161) after arrangements were made and a license was acquired from authorities in the Health Deputy of Gilan University of Medical Sciences and Rasht’s Health Center. Written consent was taken from the participants and they were assured that their personal information would remain confidential throughout the stages of data collection and entry and reporting, and that they would be disseminated only in group form.

### Data analysis

The data was analyzed in SPSS software version 23 using descriptive statistics (calculating measures of central tendency and dispersion for quantitative variables and frequency and percentage for qualitative variables). Independent t-tests, Mann-Whitney tests, one-way analysis of variance, and correlation analysis were conducted to examine the relationship between study variables. Multiple linear regression analysis was performed to predict and determine factors associated with SCB in type 2 diabetes patients. A significance level of *p* < 0.05 was considered for the tests.

## Results

The study was conducted on 341 patients with type 2 diabetes mellitus in Rasht. The mean age of the participants was 56.7 ± 9.7 years and the mean age of diagnosis was 46.1 ± 10.6 years. The shortest duration of disease was 1 year and the longest duration was 34 years. The mean duration of disease was 10.5 ± 7.1 years and the mean BMI was 29.2 ± 4.9, i.e., on average, the patients were overweight (Table 1).

**Table 1:** Demographic characteristics of participant type 2 diabetic patients in rasht

Variables	Subgroup	Frequency	Percentage
Age	30–39 Years	13	3.8
	40–49 Years	67	19.6
	50–59 Years	128	37.5
	60–69 Years	102	29.9
	70 Years and Above	31	9.1
Gender	Male	124	36.4
	Female	217	63.6
Marital Status	Married	302	88.6
	Single	3	9
	Widowed	30	8.8
	Separated	6	1.8
Educational status	Illiterate	0	0
	Primary	206	60.4
	Intermediate	84	24.6
	High School	38	11.1
	Academic	13	3.8
Occupation	Housewife	195	57.2
	Freelancer	55	16.1
	Worker	4	1.2
	Retired	42	12.3
	Employee	4	1.2
	Farmer	41	12
Age of Diagnosis	Under 30 Years	25	7.3
	30–39 Years	65	19.1
	40–49 Years	121	35.5
	50–59 Years	93	27.3
	60 Years and Above	37	10.9
Duration of disease	1–9 Years	173	50.7
	10–19 Years	112	32.8
	20–29 Years	49	14.4
	30 Years and Above	7	2.1
Oral treatment and insulin	Diet	0	0
	Oral Medications	0	0
	Insulin Injection	31	9.1
	Diet And Oral Medications	310	90.9
Income	Less Than 5 million Tomans	139	40.8
	More Than 5 million Tomans	202	59.2
BMI	18.5–24.99	60	17.6
	25–29.99	153	44.9
	30–34.99	84	24.6
	35–39.99	33	9.7
	40≤	11	3.2
Training History	No	106	31.1
	Yes	235	68.9

Table 1 shows that 124 (36.4%) patients were male and 217 (63.6%) were female. Most of the patients were married (88.6%) and had primary education (60.4%). Moreover, most of them were homemakers (57.2%). The most common age of diagnosis was observed in the 40–49 age group (35.5%) and the longest duration of disease was 1–9 years (50.7%). The majority of patients managed their disease through both diet and oral medications (90.9%), and the remaining injected insulin (9.1%). Fifty-nine-point-2% (59.2%) of the patients had incomes greater than 5 million Tomans a month, and the remaining had incomes smaller than this amount. The majority of the patients were 1st to 3rd degree overweight (82.4%). Mostly, they had no history of training (68.9%) and 31.1% had been trained by physicians, healthcare providers and health workers.

According to Table 2, the mean and standard deviation of SCB were 3.3 ± 0.89 (average self-care), those of HDSCR were 27.3 ± 7.2 (average self-care needs), and were 2.4 ± 0.45 for overall self-efficacy (average self-efficacy) (Table 2).

**Table 2:** Descriptive indices of self-care behaviors, health deviation self-care requisites, and self-efficacy in participant type 2 diabetic patients in rasht

Domains	Values (mean ± standard deviation)	Lowest and highest scores attained	Status
Self-care behaviors	3.3 ± 0.89	0.54–5.6	Average
Health deviation self-care requisites	27.3 ± 7.2	7–49	Average
Self-efficacy	2.4 ± 0.45	1.25–3.75	Average

The statistical tests ANOVA, T-test, and Mann-Whitney indicated no significant relationships between the mean scores of SCB in different age groups (*p* = 0.539), in two gender groups (male and female) (*p* = 0.203), by marital status (*p* = 0.595), educational status (*p* = 0.896), occupation (*p* = 0.247), duration of illness (*p* = 0.7), Oral treatment and insulin (*p* = 0.701), income level (*p* = 0.276), and BMI (Body Mass Index) (*p* = 0.083), and the distributions of the mean scores of SCB were similar across these groups. However, there was a significant relationship between the mean scores of SCB based on the age of diagnosis (*p* = 0.26) and the training history (*p* < 0.001). In other words, patients with a training history had significantly higher mean scores of SCB than patients without a training history.

Table number 3 shows the mean scores for 5 domains of SCB and self-efficacy. Among the five domains of self-care behavior, the highest score was related to medication compliance, with a mean score of 5.8 ± 1.3 (proper self-care), and the lowest score was related to blood sugar control, with a mean score of 0.69 ± 1.2 (poor self-care). Among the five dimensions of self-efficacy, self-efficacy in medication compliance had the highest mean score of 3.2 ± 0.45 (proper efficacy), and the lowest scores were related to self-efficacy in physical activity with a mean score of 1.8 ± 0.98 and self-efficacy in blood sugar control with a mean score of 1.8 ± 0.99 (poor self-efficacy) (Table 3).

**Table 3:** A comparison of the self-care behaviors and self-efficacy domains’ mean values

Domains	Subgroup	Values (mean ± standard deviation)	Lowest and highest scores attained	Status
Self-care behaviors	Dietary adherence	3.4 ± 1.5	0–7	Average
	Physical activity	1.6 ± 2.1	0–7	Poor
	Blood glycemic control	0.69 ± 1.2	0–7	Poor
	Foot care	4.3 ± 1.4	0–7	Average
	Medication compliance	5.8 ± 1.3	0–7	Proper
Self-efficacy	Dietary adherence	2.2 ± 0.91	1–4	Average
	Physical activity	1.8 ± 0.98	1–4	Poor
	Blood glycemic control	1.8 ± 0.99	1–4	Poor
	Foot care	2.9 ± 0.74	1–4	Proper
	Medication compliance	3.2 ± 0.45	1–4	Proper

Table number 4 displays the correlation coefficients between the scores of SCB and its domains with HDSCR and the self-efficacy constructs of type 2 diabetes patients. There was a strong and direct linear correlation between SCB and the scores of dietary adherence behavior (*r* = 0.731), self-efficacy construct (*r* = 0.689), and foot care (*r* = 0.606). There was a moderate and inverse correlation between SCB and the scores of HDSCR (*r* = 0.545), a moderate and direct correlation with physical activity behavior (*r* = 0.527), and a weak and direct correlation with medication compliance (*r* = 0.431) and blood sugar control (*r* = 0.345). In all cases, these associations were statistically significant (Table 4).

**Table 4:** Correlation matrices of self-care behaviors and its domains, health deviation self-care requisites, and self-efficacy in type 2 diabetic patients in rasht

	Self-Care Behaviors	Dietary Adherence	Physical Activity	Blood Glycemic Control	Foot Care	Medication Compliance	Health Deviation Self-Care Requisites	Self-Efficacy
Self-Care Behaviors	1	-	-	-	-	-	-	-
Dietary Adherence Behavior	0.731 ** < 0.001	1	-	-	-	-	-	-
Physical Activity Behavior	0.527** < 0.001	0.140** 0.010	1	-	-	-	-	-
Blood Glycemic Control Behavior	0.345** < 0.001	0.124* 0.022	0.058 0.286	1	-	-	-	-
Foot Care Behavior	0.606** < 0.001	0.198** < 0.001	0.173** 0.001	0.172** 0.001	1	-	-	-
Medication Compliance Behavior	0.431** < 0.001	0.260** < 0.001	0.101 0.063	0.120* 0.027	0.188** < 0.001	1	-	-
Health Deviation Self-Care Requisites	■-0.545** < 0.001	-0.303** < 0.001	-0.360** < 0.001	-0.259** < 0.001	-0.388** < 0.001	-0.278** < 0.001	1	-
Self-Efficacy	0.689** < 0.001	0.463** < 0.001	0.393** < 0.001	0.372** < 0.001	0.408** < 0.001	0.349** < 0.001	0.538** < 0.001	1

■ Pearson, and the rest were Spearman

** *p* < 0.01

* *p* < 0.05

Table 5 shows multiple linear regression results for the prediction of SCB among type 2 DM patients based on the HDSCR and the self-efficacy constructs. The physical exercise construct of self-efficacy -based on the standardized coefficient (0.311) was the most important predicting variable in the model. A unit increase in the physical exercise construct of self-efficacy score was associated with a 0.311 unit increase in the mean self-care behavior score, which was significant. With a standard coefficient of 0.239, the foot care construct of self-efficacy was the next strongest predictor of SCB. A unit increase in the foot care construct of self-efficacy score was associated with a significant 0.239 unit increase in the mean self-care behavior score. The HDSCR construct (0.209) also exhibited significant associations with SCB independently. The diet construct (standard coefficient of 0.204) ranked next in terms of predicting the distribution of SCB. With a standard coefficient of 0.184, the medication compliance construct of self-care ranked next in its power of predicting the distribution of SCB. The coefficient for determining the final model was calculated at 0.542 (Table 5).

**Table 5:** Regression analysis of predictive factors of self-care behaviors based on the health deviation self-care requisites and self-efficacy

Variable	β	Standard error	Standardized β	t	***p***-value	Lower boundary	Upper boundary
Permanent Number	0.005	0.187		0.028	0.977	-0.363	0.373
Health Deviation Self-Care Requisites	-0.026	0.006	-0.209	-4.596	< 0.001	-0.015	-0.037
Self-Efficacy in Diet	0.201	0.038	0.204	5.292	< 0.001	0.126	0.276
Self-Efficacy in Physical Activity	0.284	0.036	0.311	7.982	< 0.001	0.214	0.354
Self-Efficacy in Blood Glycemic Control	0.125	0.036	0.138	3.434	0.001	0.053	0.197
Self-Efficacy in Foot Care	0.288	0.049	0.239	5.920	< 0.001	0.192	0.383
Self-Efficacy in Medication Compliance	0.216	0.045	0.184	4.791	< 0.001	0.127	0.304

*R*_2_=0.542

## Discussion

Findings from a cross-sectional study to assess the status of SCB, self-efficacy, and DSCR based on Orem’s model in type 2 diabetes patients in Rasht, Iran discussed from two points of view.

### The statues of SCB in type 2 diabetes patients

The results of this study also demonstrated that type 2 diabetes patients had average self-care scores (47.1% of the overall score). This too is consistent with many other studies in the literature [33–35]. Though, Kong et al. [22] reported SCB at a high level among type 2 diabetes patients, and Abate et al. [36] and Emire et al. [25] reported them at a low level, all of which are in contrast to our findings. The differences may be due to different study populations, varying levels of awareness and patients’ attitudes toward self-care and/or the use of different tools. Furthermore, extensive training offered through the media can lead to different SCB among patients in different countries and even various regions of a single country.

### Association between demographic characteristics and self care behaviors in patients with type 2 diabetes patients

Our findings showed that most of our patients were female, and women had higher SCB scores than men. However, gender did not significantly affect SCB, which agreed with many other studies [23, 33]. In contrast, Nejat et al [34] and Kong et al. [22] found significant associations between gender and SCB, which differed from our results. This discrepancy might be explained by the variation in gender inequality among individuals. Moreover, other variables, such as education, physical, psychological and cultural factors, could also influence the impact of gender on SCB. Most of the participants were married, and their marital status did not significantly influence SCB, which was consistent with previous studies [23, 33]. This suggested that SCB depended on various factors besides marital status. However, Abate et al. [36] and Modaresi et al. [37] reported significant associations between marital status and SCB, which contradicted our findings. This inconsistency might be due to the differences in the sample characteristics and the proportion of single and married patients. According to our results, most of the participants were homemakers, and their occupation did not significantly affect SCB. This was similar to the findings of many other studies [23]. However, Kong et al. [22] and Abate et al. [36] demonstrated that occupation significantly influenced SCB, which opposed our findings. This divergence might be attributed to the variations in the definitions of occupations across these studies and the relationships between these occupations and confounding and influential variables such as income, education, etc. The majority of the participants were in the 50-59-year-old age group, and the highest self-care score was in the 40-49-year-old age group. The self-care scores decreased with increasing age, but the difference was not statistically significant among the age groups in terms of SCB. This was in line with Modaresi et al’s [37] findings. In the present study, there was no significant association between SCB and duration of disease; longer disease duration did not result in better SCB in patients. This was consistent with Ishak et al’s [33] findings. Although patients became more experienced and empowered in self-care over time, the chronicity of the disease caused physical and psychological complications and reduced the patient’s adherence to self-care activities. However, Modaresi et al. [37] found a significant association between SCB and duration of disease in diabetic patients, which differed from our findings. This difference might be explained by the shorter duration of disease (< 1 year) and the lower mean age of the participants (2.2 years) in Modaresi’s study. Furthermore, the effects of confounding and influential variables such as knowledge, awareness, etc. might also be involved.

Our findings indicated that most of our participants had high school education (96.2%) and only 3.8% had academic education. Those with higher education levels showed more SCB than those with lower education levels, suggesting that education was a determinant factor of SCB in patients. Ebrahimi et al. [38] argued that higher education enhances decision making, self-confidence and self-care. However, we did not find a significant association between education and self-care scores in this study, which might be due to the unequal distribution of participants across different education levels. Our results were consistent with those of other studies [23, 34]. In contrast, Abate et al. [36] reported a significant association between SCB and education, which differed from our findings. This discrepancy might be explained by the large number of illiterate participants in Abate’s study, while there were none in our study. Most of our participants (90.9%) followed a Oral treatment and insulin, while 9.1% used insulin injections. The self-care scores of these two groups did not differ significantly. This was in line with our results, Ishak et al. [33] also did not find a significant association between SCB and treatment type -with or without insulin-. However, Modaresi et al. [37] found a significant relationship between SCB and treatment type, which opposed our findings. This difference might be due to the different assessment tools or patient characteristics, such as in Modaresi’s study, 45.7% of patients used insulin injections, while only 9.1% did so in our study, and the rest used diet and oral medications. In the present study, like other studies [22, 33], most of the overweight patients (BMI > 25) (82.4%) had lower self-care scores than those with normal BMI (18–25), but this difference was not statistically significant. On the other hand, Modaresi et al. [37] detected a statistically significant association between BMI and self-care status. The oldest age of diagnosis in our study was in the 40-49-year-old age group. The self-care scores at ages of diagnosis below 30 years and over 60 years were lower than the other age groups, and this difference was statistically significant. This might indicate that younger individuals have lower tendencies toward self-care due to their higher risk-taking and curiosity for new experiences. In the elderly over 60 years, lower levels of awareness, loss of abilities, reduced activity and mobility, loss of friends and loved ones, decreased financial and physical independence, and chronic diseases might account for their lower self-care scores compared to the other age groups. Furthermore, this study showed that patients who had received training had higher self-care scores than those who had not, and this difference was statistically significant. Our finding agreed with those of Chali et al. [24]. Therefore, it seems that education/training can significantly improve patients’ SCB.

### Correlation of self-care behaviors and its domains

Our findings showed a strong correlation between SCB and self-efficacy, which agreed with many other studies. Rahaee et al [23], Kong et al. [22] and Chali et al. [24] found significant direct associations between SCB and self-efficacy. This means that individuals with higher self-efficacy set bigger goals and expect better outcomes; they view the challenges of self-care as solvable problems and adopt SCB. However, some studies did not find any association between self-efficacy and SCB. Chlebowy et al. [27] reported no significant relationship between self-efficacy and self-care. These differences might be due to the use of different assessment tools and the diversity of study populations and cultures. SCB had strong correlations with diet adherence and foot care; moderate correlations with physical activity; and weak correlations with medication compliance and glycemic control. SCB also had a moderate and negative correlation with HDSCR; that is, higher patient needs were associated with lower self-care. Among the self-care components, there was a strong correlation between dietary adherence and medication compliance. In addition, various components of SCB were statistically and significantly correlated with diet adherence, medication compliance, foot care, physical activity, and glycemic control. The following pairs had statistically significant correlations: physical activity and foot care, glycemic control and foot care and medication compliance, and foot care and medication compliance. However, physical activity was not significantly correlated with medication compliance and glycemic control. Physical inactivity is sometimes habitual, and in some cases, age and awareness of the role of physical activity can affect disease control; starting a regular exercise program requires ongoing training and a suitable environment [39].

### The predictability of type 2 diabetic patients’ SCB

Multiple linear regression was used to determine the predictability of self-efficacy and HDSCR in SCB. The current study’s results highlight the importance and predictive effect of HDSCR in self-efficacy, particularly exercise self-efficacy, foot care self-efficacy, and dietary self-efficacy in type 2 diabetic patients’ SCB. This model showed that 54% of the changes related to SCB in these patients can be explained by self-efficacy and HDSCR.

### Generalizability of findings

In our study, we ensured generalizability by carefully selecting a representative sample from the population of interest. Specifically, we employed random sampling from 32 rural health houses in Rasht city, selecting 27 one with suitable geographical distribution. We evaluated 1125 patients out of 1809 with active profiles registered in these centers based on our inclusion criteria. Furthermore, our statistical analysis was robust, enhancing the external validity of our findings. While our methodology was designed to ensure the results are not confined to the specific sample, we caution against over-generalization and recommend further studies for broader applicability.

### Implication of findings

The implications of our study findings for research and practice are as follows:

#### For research

Our study provides valuable insights into the complex relationship between self-care, self-efficacy, and Health Deviation Self-Care Requisites in patients with type 2 diabetes. This contributes to the existing knowledge base and opens avenues for further research to explore these relationships in more depth. Moreover, the strong and direct correlation between self-care behaviors and self-efficacy, as well as the role of the physical exercise construct of self-efficacy as a significant predictor of self-care behaviors, are key findings that could guide future research in this area.

#### For practice

The findings underscore the importance of addressing self-efficacy and specific self-care domains, such as physical activity and foot care, in diabetes management strategies. The results indicate that self-efficacy is a dynamic and changeable belief that can be influenced by behavioral interventions. This suggests that teaching self-care behaviors is essential in promoting self-efficacy and empowering patients. The study may inform healthcare professionals and policymakers in developing targeted interventions to improve self-care practices in diabetic patients. Specifically, purposeful and needs-based training grounded on promotion models that identify the determinant factors of self-care are necessary to overcome behavioral barriers.

### Limitation

The limitations of the study can be discussed in terms of potential sources of bias and imprecision. The study has several limitations that could introduce bias or imprecision. Firstly, the participant selection was limited to rural patients attending a comprehensive health center, which may not represent the broader population. This could introduce selection bias, potentially skewing the results in an unknown direction. Secondly, there was a gender imbalance with a greater number of female participants compared to males. This could lead to gender bias if the outcomes measured are differentially affected by gender. The direction of this bias is uncertain and could either overestimate or underestimate the true effect size. Thirdly, the lower educational level of the participants was addressed by having the researcher complete the questionnaires. While this approach reduces the risk of information bias due to misunderstanding of questions, it could introduce observer bias if the researcher unconsciously influences the responses. Lastly, the data were self-reported, which may introduce recall bias or social desirability bias. Participants may not accurately remember past events (recall bias) or may answer in a way that they believe is socially acceptable rather than truthful (social desirability bias). Both biases could lead to an overestimation or underestimation of the true effect size. However, while these limitations could potentially bias the results, the direction and magnitude of the bias are uncertain. Future studies should aim to address these limitations to provide more accurate and generalizable results.

## Conclusions

Our results indicated that type 2 diabetic patients’ SCB in Rasht were undesirable and that their demographic traits did not play influential roles in this regard. As one of the most important determinants of SCB, self-efficacy is of paramount importance in diabetic patients. Self-efficacy is a dynamic and changeable belief, and can change with behavioral interventions. Therefore, teaching SCB is essential in promoting self-efficacy and empowering patients. In fact, to promote patients’ SCB, purposeful and needs-based training grounded on promotion models that identify the determinant factors of self-care are necessary to overcome behavioral barriers.

## Data Availability

The datasets utilized and/or analyzed in the present study can be obtained from the corresponding author upon reasonable request.

## Abbreviations

SCB: Self-Care Behaviors
HDSCR: Health Deviation Self-Care Requisites
WHO: World Health Organization
BMI: Body Mass Index
